# Reactive Oxygen Species Imaging in a Mouse Model of Inflammatory Bowel Disease

**DOI:** 10.1007/s11307-016-0934-0

**Published:** 2016-02-12

**Authors:** Laura Bronsart, Linh Nguyen, Aida Habtezion, Christopher Contag

**Affiliations:** Department of Biology, Stanford University, 318 Campus Drive, Stanford, CA 94305 USA; Department of Pediatrics, Stanford University, 318 Campus Drive, Stanford, CA 94305 USA; Division of Gastroenterology and Hepatology, Department of Medicine, Stanford University, 318 Campus Drive, Stanford, CA 94305 USA; Departments of Radiology, Microbiology & Immunology, Stanford University, 318 Campus Drive, Stanford, CA 94305 USA

**Keywords:** Superoxide anion, Reactive oxygen species, Inflammatory bowel disease, Coelenterazine, Imaging

## Abstract

**Purpose:**

Reactive oxygen species (ROS) are important contributors to inflammatory bowel disease (IBD); however, there are insufficient tools for their *in vivo* evaluation.

**Procedures:**

To determine if a chemiluminescent ROS reporter, coelenterazine, would be a useful tool for the detection of immune cell activation, the macrophage cell line (RAW 264.7) was treated with phorbol myristate acetate (PMA). Additionally, coelenterazine was used to monitor the changes in ROS production over time in a mouse model of IBD.

**Results:**

*In vitro*, coelenterazine enabled the dynamic monitoring of the RAW 264.7 cell oxidative burst. *In vivo*, there were early, preclinical, changes in the localization and magnitude of coelenterazine chemiluminescent foci.

**Conclusions:**

Coelenterazine offers a high-throughput method for assessing immune cell activation in culture and provides a means for the *in vivo* detection and localization of ROS during IBD disease progression.

## Introduction

Uncontrolled and chronic inflammation can be an insidious disease, often developing and progressing substantially prior to the onset of clinical signs. This is especially apparent in animal models of disease where clinical signs are often masked by protective animal behaviors. For these reasons, animal research has primarily been focused on late-stage, symptomatic disease. Importantly, this has limited our understanding of disease pathogenesis as it relates to humans and has, therefore, prevented the development of optimized therapeutics. Imaging technologies for the *in vivo* detection of subclinical inflammation would be a useful aid in advancing research in these fields.

Currently, there are few methods for detecting subclinical inflammation in mouse models of disease. Molecular imaging techniques that utilize bioluminescence are the most efficient method for detection of disease states; however, their use has been primarily limited to the detection of engineered cell lines, cell and tissue transplant models, or transgenic mice. This is because bioluminescence imaging requires the introduction of a luciferase enzyme-encoding gene into the cell line or donor animal of interest [1–5]. A similar technique, chemiluminescence imaging, relies on the interaction between a small molecule and a biological substrate, which may be intrinsic to the tissue, and detection of the resultant photon emission. Although chemiluminescent signals are generally less intense than bioluminescent sources of light, chemiluminescence imaging does not necessitate the use of transgenic cell lines or animals [6]. Identification of appropriate chemiluminescent reagents that react with inflammation-associated molecules would enable early detection of subclinical inflammation in animal models and support research aimed at early disease pathogenesis.

**Fig. 1:**
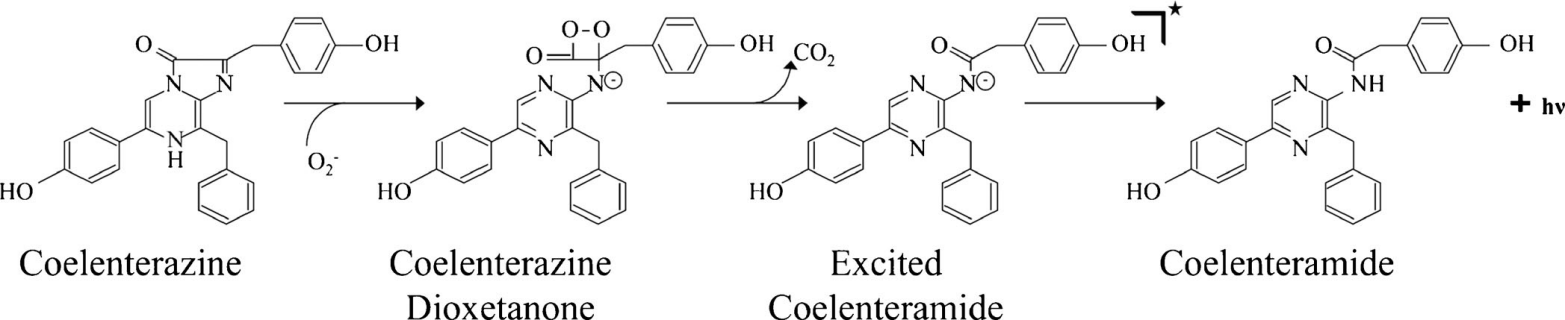
The reaction of coelenterazine with superoxide anion resulting in the production of chemiluminescence.

Among the molecules associated with inflammation are reactive oxygen species (ROS); local and significant increases in ROS concentrations have been associated with inflammatory responses [7]. Inflammatory cells produce and release ROS into the extracellular space as part of the respiratory burst [8–12]. These ROS, including superoxide anion and peroxynitrite, have antimicrobial effects due to damage to the proteins, lipids, and DNA of target cells [13, 14]. ROS concentrations have been shown to be greater at sites of active inflammatory bowel disease (IBD) in excised tissues [15–18]. Additionally, treatment with ROS scavengers has been shown to be protective in some IBD models [18, 19]. Despite this, fluctuations in IBD-associated ROS have yet to be longitudinally studied in animals due to a lack of *in vivo* ROS reporters. The small molecule coelenterazine is known to produce chemiluminescence upon reaction with superoxide anion and peroxynitrite (Fig. 1) [20, 21]. Coelenterazine has been shown to detect the neutrophilic oxidative burst *in vitro* [22]. Additionally, *in vivo* delivery of coelenterazine is considered safe; it is the substrate for the Gaussia and Renilla luciferases and is frequently administered for *in vivo* bioluminescence imaging [1, 23–25]. As such, we tested if coelenterazine ROS-mediated chemiluminescence would be useful for the detection of IBD-associated ROS in the dextran sodium sulfate mouse model.

## Materials and Methods

### Materials

Native, i.e., unmodified, coelenterazine was purchased from NanoLight (#303).

### Cell Culture

RAW 264.7 cells were cultured in DMEM with 10 % fetal bovine serum (Invitrogen), 100 U/ml penicillin, and 100 mg/ml streptomycin.

### Live Cell Imaging

RAW 264.7 cells were split 48 h prior to the experiment to achieve a final confluency of approximately 60 % the day of the experiment. The day of the experiment, cells were collected by mechanical disruption. Following collection, cells were washed twice in PBS and resuspended in Krebs buffer (140 mmol/l NaCl, 30 mmol/l Hepes, 4.6 mmol KCl, 1 mmol/l MgSO_4_, 0.15 mmol/l Na_2_HPO_4_, 5 mmol/l NaHCO_3_, and 2 mmol/l CaCl_2_) supplemented with 6 mmol/l d-glucose. Cells were counted and 5 × 10^4^ cells were added to wells of a 96-well black plate. All samples were completed in triplicate. Native coelenterazine was added to each sample to achieve a final concentration ranging from 0 to 100 μmol/l. The plate was imaged using an IVIS (Xenogen line of products from Perkin Elmer) low-light imaging system. Post-imaging, the wells were treated with 1.6 μmol/l of phorbol myristate acetate (PMA) or equal-volume vehicle control and imaged immediately and sequentially for 60 min. The total flux (p/s) was quantified for each well using the Living Image software.

### Ethics Statement

All animal studies were completed in accordance with the Guide for the Care and Use of Laboratory Animals of the National Institutes of Health and approved by the Institutional Animal Care and Use Committee of Stanford University.

### IBD Animal Model

#### Dextran Sulfate Sodium-Induced Colitis

Inflammatory bowel disease was induced in 5 male, BALB/c, age 6 weeks, mice using the dextran sulfate sodium (DSS) model as previously described [26]. Briefly, the drinking water of experimental animals was replaced with an aqueous solution of 2 % DSS on day 0. The mice were monitored daily for changes in body condition, the presence of bloody stools, and general morbidity. The animals were euthanized at or prior to a 20 % reduction in body weight.

#### Mouse Imaging

Mice were imaged on day 0 prior to the supplementation with DSS and every 48 h thereafter for 10 days. Mice were anesthetized using isoflurane inhalant anesthetic and administered 5 mg/kg native coelenterazine intravenously. Mice were immediately imaged using the IVIS Spectrum optical imaging platform and the data quantified using Living Image software. A region of interest (ROI) encompassing each entire mouse was selected, and the data was presented as the total flux (p/s).

### Statistical Analysis

Data are presented as the mean ± SEM. Significance was determined by Student’s two-tailed *t* test and defined as *P* ≤ 0.05.

## Results

### RAW 264.7 Cells Induce Coelenterazine Chemiluminescence in Culture

**Fig. 2:**
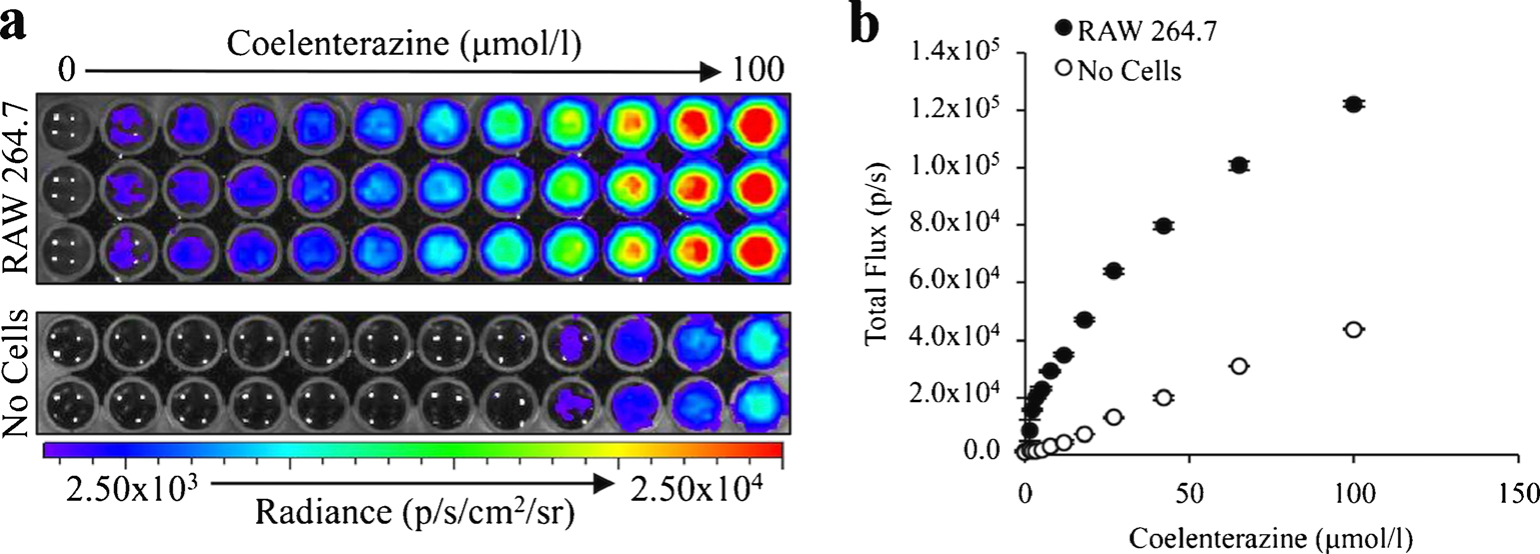
RAW 264.7 chemiluminescent signal intensity positively correlates with coelenterazine concentration in culture. **a** 5 × 10^5^ RAW 264.7 cells or a buffer control (no cells) were imaged following the addition of native coelenterazine ranging in concentration from 0 to 100 μmol/l. **b** Quantification of coelenterazine chemiluminescence (*n* = 3) (*error bars* represent the mean ± SEM).

We have previously demonstrated that non-immunological cell lines induce coelenterazine-mediated chemiluminescence under basal conditions [27], but it was unknown if the macrophage cell line, RAW 264.7, would have a similar response. To evaluate this, RAW 264.7 cells were exposed to coelenterazine concentrations ranging from 0 to 100 μmol/l and imaged using a CCD camera (IVIS). There was detectable chemiluminescence that was dependent upon coelenterazine concentration (Fig. 2a). The cellular-dependent chemiluminescence became significantly greater (*P* ≤ 1.29 × 10^−6^) than the buffer-mediated chemiluminescence at a minimum concentration of 2 μmol/l coelenterazine. No plateau in signal intensity was observed (Fig. 2b).

**Fig. 3:**
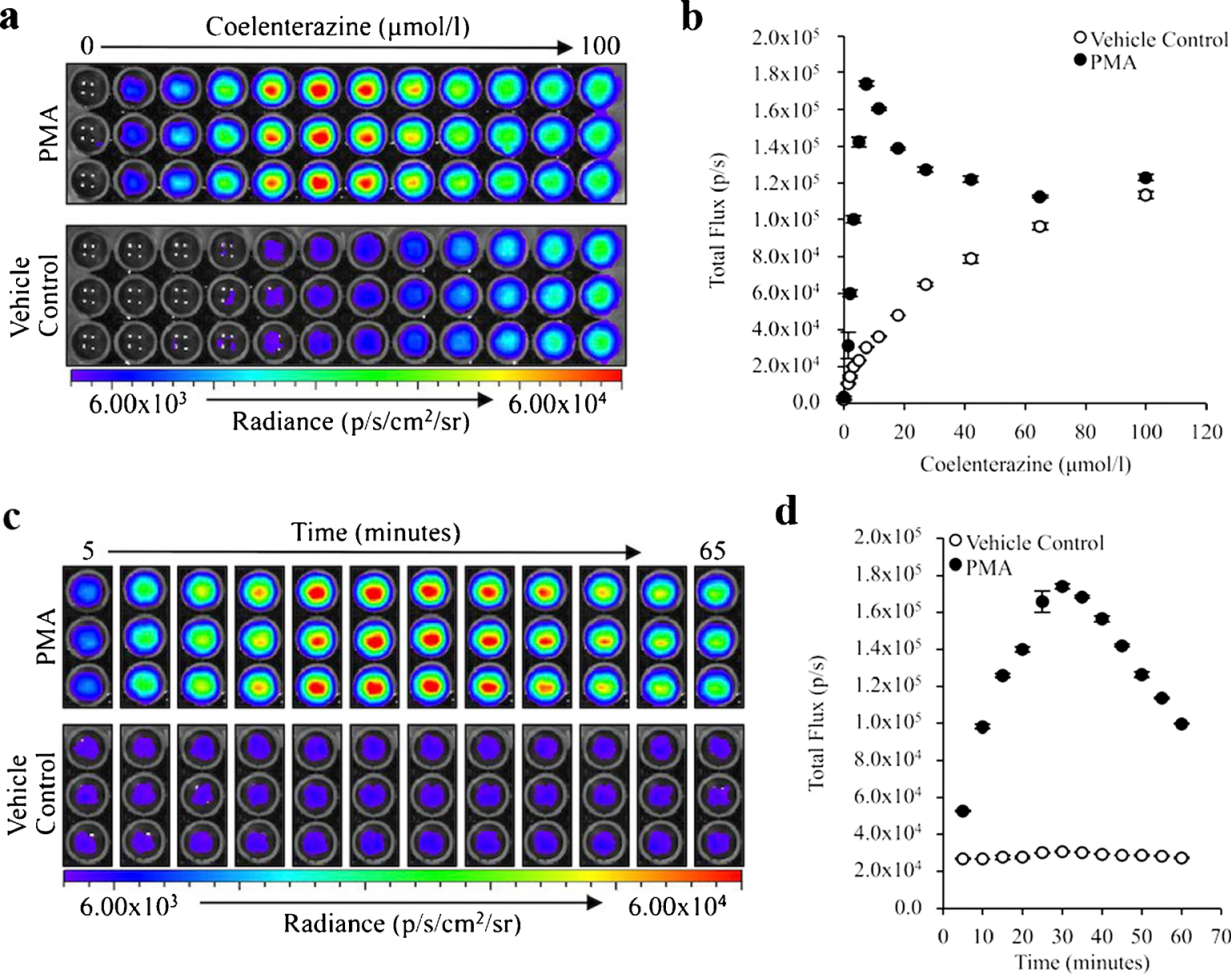
RAW 264.7 stimulation is detectable using coelenterazine. **a** Chemiluminescent image of 5 × 10^5^ RAW 264.7 cells incubated with 0 to 100 μmol/l of native coelenterazine 30 min following the addition of 1.6 μmol/l phorbol myristate acetate (PMA) or vehicle control. **b** Quantification of coelenterazine chemiluminescence (*n* = 3). **c** Sequential chemiluminescent images of 5 × 10^5^ RAW 264.7 cells incubated with 7.5 μmol/l of native coelenterazine and treated with 1.6 μmol/l of PMA or vehicle control. **d** Quantification of coelenterazine chemiluminescence (*n* = 3) (*error bars* represent the mean ± SEM).

The production of chemiluminescence by RAW 264.7 cells in the absence of stimulation is consistent with the detection of superoxide anion production during oxidative phosphorylation. To determine if coelenterazine would be useful for detecting inflammation-associated reactive oxygen species, we next assessed the signal intensity produced by RAW 264.7 cells stimulated with phorbol myristate acetate (PMA). PMA, a protein kinase C activator, has been shown to induce a respiratory burst in macrophages [8]. RAW 264.7 cells were incubated with increasing concentrations of coelenterazine and then treated with PMA and imaged serially. The ability to detect a cellular response to PMA was dependent upon the concentration of coelenterazine. At the highest concentration of coelenterazine, no difference was detectable between cells treated with PMA and those treated with a vehicle control (Fig. 3a). The maximal difference between PMA-treated and control cells was achieved with a coelenterazine concentration of 7.5 μmol/l (Fig. 3b). The coelenterazine concentration of 7.5 μmol/l enabled sequential imaging and quantification of the cellular response to PMA stimulation (Fig. 3c). Significant signal (*P* ≤ 7.92 × 10^−7^) was detected within 5 min of PMA treatment with the peak signal intensity being achieved 30 min after treatment and declining after that point (Fig. 3d). Based on these results, coelenterazine enabled high-throughput, sequential imaging and quantification of reactive oxygen species production in live, cultured macrophages

### Coelenterazine Detects Reactive Oxygen Species Production in Response to IBD

**Fig. 4:**
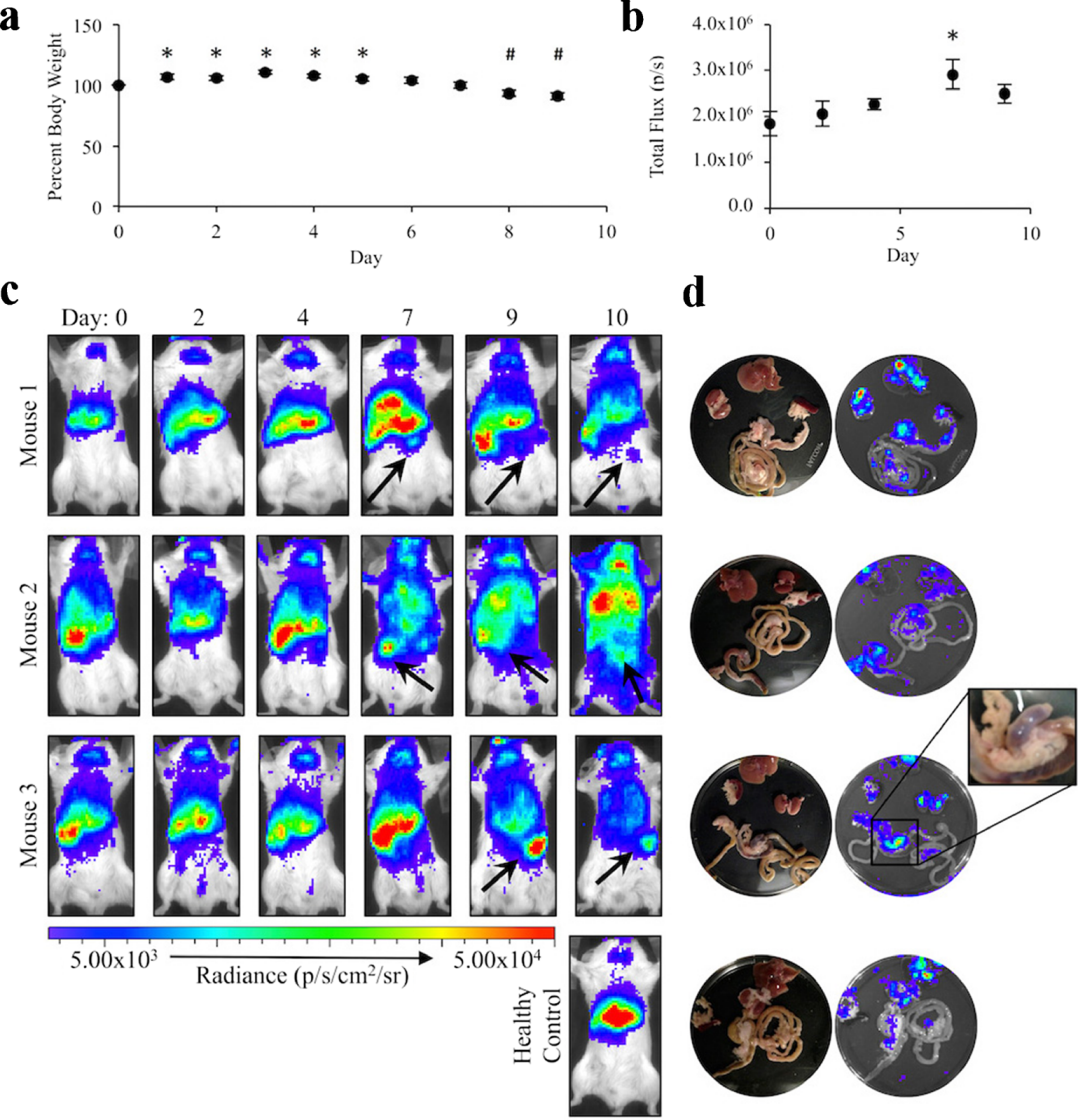
Coelenterazine detects changes in regional reactive oxygen species production in response to IBD. **a** Percent body weights relative to day 0 (*n* = 5). **b** The average chemiluminescent signal intensity quantified as the total flux (p/s) for dextran sulfate sodium (DSS)-treated mice imaged using coelenterazine (*n* = 4). **c** Chemiluminescent images of three representative mice in **b** prior to treatment initiation (day 0) and every 48 h thereafter. A healthy mouse was imaged on day 10 for comparison. *Arrows* indicate new regions of chemiluminescent signal generation. **d** Chemiluminescent and white light images of the abdominal contents of the mice in **b/c** (*error bars* represent the mean ± SEM. *Significant increase relative to day 0 with *P* < 0.04; ^#^significant decrease relative to day 0 with *P* < 0.04).

Having confirmed that coelenterazine is a useful agent for the detection of reactive oxygen species produced by macrophages in response to activation in culture, we next aimed to determine if it could detect changes in reactive oxygen species production *in vivo* during an inflammatory disease. Mice were imaged prior to disease induction with DSS and every 48 h thereafter. On day 8 of the model, the average body weight of the mice was significantly decreased (*P* = 0.0333) at 93.4 ± 2.6 % (mean ± SEM) of the starting body weight (Fig. 4a). There was substantial upper abdominal and thoracic signal that was consistent with our previous finding that the lung and the pancreas have elevated concentrations of ROS compared to other tissues [28]. Despite the signal interference, all mice developed changes in the intensity and location of chemiluminescent signal by day 7, prior to the significant reduction in body weight (Fig. 4b–c). Upon removal and imaging of the abdominal contents, chemiluminescence was not observed along the bowel wall but was evident within the mesentery associated with the colon and large intestine (Fig. 4d). This suggests that the greatest amount of reactive oxygen species is produced, not within the bowel wall, but within the associated mesenteric lymphatic tissue at this stage of disease.

## Discussion

The small molecule coelenterazine is known to react with the reactive oxygen species superoxide anion and peroxynitrite with resultant chemiluminescence [20]. This quality had been used to detect the respiratory burst of neutrophils using a luminometer; however, it was unknown if the signal intensity was sufficient for live cell imaging in culture or for *in vivo* imaging of inflammation [22].

The goal of this study was to determine if coelenterazine would be a useful tool for detecting the early stages of inflammation in a mouse model of inflammatory bowel disease. We began by assessing if coelenterazine could be used to image the oxidative burst of live RAW 264.7 cells in culture. We then applied its use to the DSS inflammatory bowel disease mouse model. The results demonstrate that the imaging technique is sensitive enough to detect inflammation-mediated coelenterazine chemiluminescence in live, cultured cells *in vivo*, and that inflammatory bowel disease is marked by an early inflammatory response that precedes the development of a reduction in body weight by 24 h.

### Coelenterazine Imaging Provides a Dynamic Read-Out of the RAW 264.7 Oxidative Burst

Coelenterazine chemiluminescence imaging enabled the detection of the oxidative burst of RAW 264.7 cells in response to PMA stimulation. The dynamic nature of chemiluminescent imaging provided the means to longitudinally detect superoxide anion concentrations with fine temporal resolution in a cell culture model. Additionally, this technique supported high-throughput analysis of whole cell populations.

### In vivo Coelenterazine Imaging Detects Pre-clinical Inflammation in IBD

Mice imaged with coelenterazine demonstrated visible variations in the chemiluminescent signal pattern over the course of disease. The shift in chemiluminescent foci and intensity occurred 24 h prior to a significant reduction in body weight, the current primary method used to confirm the presence of IBD in mouse models [26]. These results indicated that inflammatory processes occur significantly earlier than the development of IBD clinical signs. These findings are supported by longitudinal studies of primary sclerosing cholangitis patients which determined that histological evidence of inflammation precedes the development of clinical signs in human patients by as much as 7 years [29]. Further understanding of this early pathology may provide clues into the progression of IBD and aid in the development of biomarkers for pre-symptomatic detection of disease and relapses. Additionally, excision of the tissues showed the chemiluminescence originated from the mesenteric-colon border, appearing to be associated with lymphatic tissues in the region. Such findings suggest that although inflammatory bowel disease is characterized as inflammation of the bowel wall, there is substantial if not major involvement of the mesentery in the inflammatory disease process.

## Conclusion

In conclusion, chemiluminescent reporters, such as coelenterazine, provide dynamic, longitudinal quantification with fine temporal resolution of ROS in populations of live, cultured cells. Additionally, coelenterazine can be used to detect early, pre-symptomatic inflammation in a mouse model of inflammatory bowel disease, making it a useful tool for studies involving early disease pathogenesis or intervention. The evidence for early changes in ROS production suggests that novel ROS reporters, that can be translated to the clinic, may provide an avenue for the use of ROS detection in disease monitoring and prognostication. A fluorescent reporter of ROS could be employed when used in conjunction with fluorescent endoscopic and/or laparoscopic devices.
